# Synovial cysts of the hip joint: a single-center experience

**DOI:** 10.1186/s12893-018-0450-z

**Published:** 2018-12-05

**Authors:** Jingjing Wang, Jiang Shao, Chenyang Qiu, Yu Chen, Bao Liu

**Affiliations:** Department of Vascular Surgery, Peking Union Medical College Hospital, Peking Union Medical College and Chinese Academy of Medical Sciences, Beijing, China

**Keywords:** Synovial cyst, Lower limb edema, Surgical excision

## Abstract

**Background:**

Synovial cyst of the hip joint is a rare clinical condition in need of evidence-based guidelines for its diagnosis and management. Normally, synovial cyst of the hip joint requires no treatment, but when it intrudes into surrounding structures, various clinical symptoms appear. Because of its rarity, a symptomatic synovial cyst is often confounded with a tumor as a space-occupying lesion or with other diseases, depending on its various clinical presentations. Therefore, guidelines for the precise diagnosis and appropriate management for synovial cyst of the hip joint are required.

**Methods:**

We retrospectively studied 7 cases of symptomatic synovial cyst of the hip joint, some of which showed lower limb edema due to mass effect. We compared physical exam findings on presentation, imaging findings, and size and location of the cyst.

**Results:**

All cases were managed successfully with surgical excision. We found that, instead of the size of the cyst, the location of the cyst was an important contributor to venous compression. The recurrence rate was 0%, and some patients have significantly long follow-up of 2 years, 4 years, 6 years and 10 years, respectively.

**Conclusions:**

For symptomatic synovial cyst of the hip joint, surgical excision can successfully resolve the symptoms without recurrence. This retrospective study discusses the clinical presentations, diagnostic approaches, and surgical treatment of symptomatic synovial cyst of the hip joint, hence shedding more light on the clinical management of this condition.

## Background

Synovial cysts are common in joints such as the knee, ankle, hand, and wrist, but uncommon in the hip joint. Histologically, a synovial cyst is an extension of the joint capsule with a lining of synovial cells, and it often communicates with the adjacent joint. The cyst contains viscous, gelatinous fluid due to the presence of hyaluronic acid and other mucopolysaccharides. Most of the synovial cysts originating from the hip joint are asymptomatic, and sometimes are incidentally found by imaging or clinical inspection [1]. When a synovial cyst intrudes into surrounding structures, it causes multiple problems, including pain and swelling, peripheral nerve compression, arterial/venous compression, or even urinary symptoms [2]. This retrospective study reports 7 cases of symptomatic synovial cysts of the hip joint, five of which showed lower limb edema secondary to compression of the cyst. All were treated with surgical excision with symptom relief within one week post-surgery, and with satisfactory prognosis during follow-up.

## Methods

**Fig. 1:**
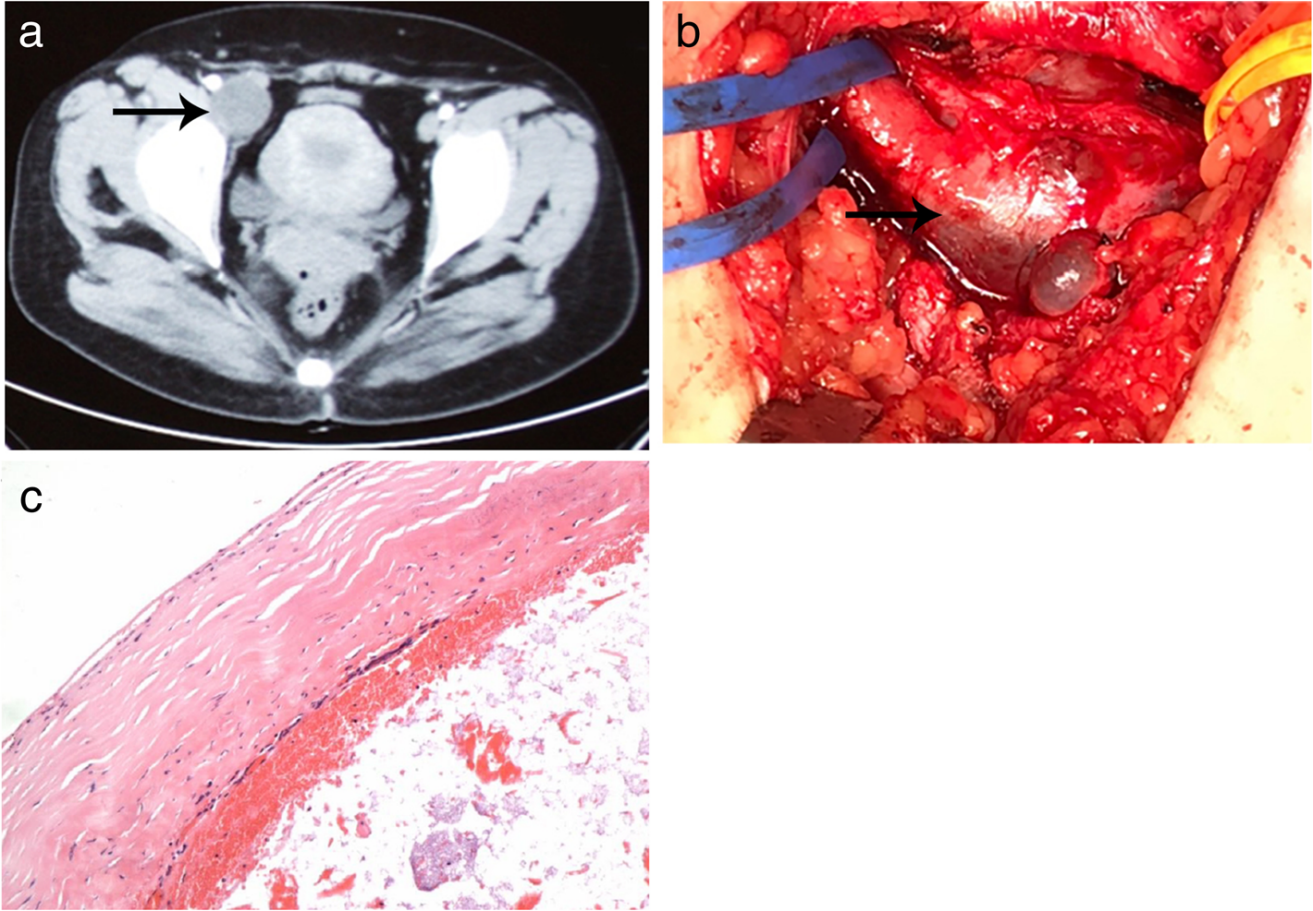
Image data and pathology result for patient NO.1. **a**. A CT showed a cystic space-occupying lesion (black arrow) in the inguinal region. **b**. At surgery, the synovial cyst (black arrow) was observed directly communicating with the hip joint. **c**. The pathology result confirmed a synovial cyst

**Fig. 2:**
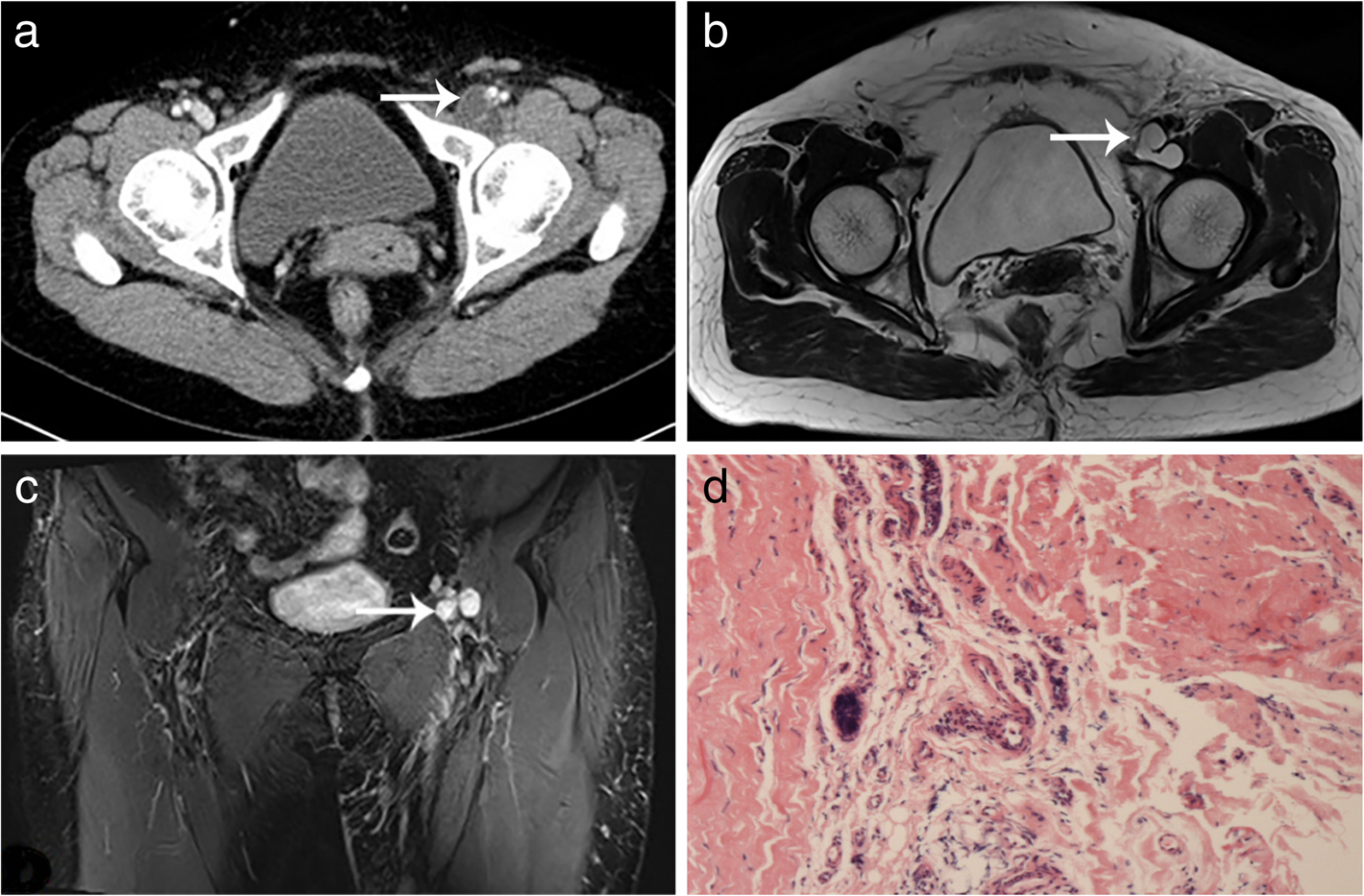
Image data and pathology result for patient NO.2. **a**. A CT and **b**. and **c**. MRI showed a cystic space-occupying lesion (white arrow) in the inguinal region, leading to severe stenosis of the common femoral vein. **d**. The pathology result confirmed a synovial cyst

We reviewed 7 cases of synovial cysts of the hip joint, which we had diagnosed from January 2000 to April 2018. These cases most commonly presented as a mass of unknown cause in the inguinal area. Some patients also showed lower limb edema in the affected side. We compared physical exam findings on presentation, imaging findings, and size and location of cyst. For all patients, no tenderness of the gastrocnemius muscle was detected, and peripheral lower extremity pulses were equal and normal bilaterally (femoral, popliteal, posterior tibial, and dorsalis pedis artery). Imaging findings were also similar in all patients. Duplex ultrasonography, conducted in all patients, did not recognize any arteriostenosis or venous embolism. Computed tomography (CT), conducted in patient NO.1–5 and 7, showed a cystic lesion around the acetabular level without enhancement (Fig. 1a and Fig. 2a). Magnetic resonance imaging (MRI), conducted in patient NO. 2 and 6, also showed a cystic lesion around the hip joint (Fig. 2b). Exploratory mass resection surgery was indicated after further evaluation of each patient, including complete blood count, liver function, kidney function, coagulation function and cardiac injury index tests. Before the surgery, no other treatment had been administered, to any of the 7 patients.

The surgical procedure was similar in each case. The patient was placed under general anesthesia; we exposed the cystic mass through the Smith-Peterson approach and carefully dissociated the mass from surrounding structures. The posterior portion of the mass was easily dissected from the hip joint capsule (Fig. 1b). Thus, the cyst was simply a herniation of the synovium into the surrounding tissue, but not a ganglion cyst that was associated with a joint or tendon sheath. The cyst was opened, revealing the yellow gelatinous liquid inside. We excised the whole cyst and then reconstructed the hip joint. Part of the cyst tissue was sent for pathological examination. Pathology results indicated a fibrocystic tissue of chronic inflammation, which confirmed the diagnosis of a synovial cyst (Fig. 1c and Fig. 2d).

Due to venous compression from the cysts, some patients required heparin and a sequential compression device as preventive measures for deep vein thrombosis. Edema often disappeared within 1 week post-surgery. Patients were discharged on oral aspirin and followed up at 1 and 6 months.

## Results

The clinical characteristics of the patients are shown in Table 1. The 7 patients in our study aged from 20 to 60 years old. One patient (case 6) had rheumatoid arthritis, and the pathology results of the cyst in this case indicated an inflammatory synovium with neovascularization and infiltration of lymphocytes and plasma cells. This suggested that rheumatoid arthritis was the underlying cause of the cyst.

**Table 1:** The clinical characteristics of the 7 cases in our study

NO.	Age range (y)^b^	Delay in diagnosis	Arthropathy	Edema	Compressed vessel	Location of the cyst^a^	Size of the mass(approx.)	Follow up
1	40–49	1 y	–	Y	CFV	right behind CFV	4 × 4 cm	9 m
2	50–59	2 m	–	Y	CFV	right behind CFV	4 × 4 cm	7 m
3	30–39	1 y	–	Y	CFV	right behind CFV	4 × 2 × 1 cm	2 m
4	40–49	1 y	–	Y (slight)	–	behind CFA,external to CFV	4 × 4 cm	4 y
5	50–59	1 y	–	N	–	behind CFA and FN	3.1 × 1.6 cm	2 y
6	20–29	9 m	RA	N	–	inside iliopsoas	5.5 × 1.4 × 1.2 cm	6 y
7	30–39	1 y	–	Y	CFV	behind CFA and FN	unknown	10 y

*y* years, *m* months, *Y* yes, *N* no, *CFV* common femoral vein, *FN* femoral nerve, *RA* rheumatoid arthritis

^a^The cysts were at the transverse level of the acetabulum. In order to simplify the table, we described the location of the cyst within that transverse level

^b^For patient’s privacy, we represented the patient’s age as age range

Five patients (cases 1, 2, 3, 4 and 7) showed edema of the lower limb due to compression of the common femoral artery by the synovial cyst, proven by both imaging techniques and observations during the surgery. Typically, these cases had many more adhesions encircling the synovial cyst and the common femoral vein. In case 1, part of the cystic wall was not resected because it could not be separated from the femoral vein. On the other hand, when edema was not present, there were no obvious adhesions, and the synovial cyst could be easily dissociated by blunt dissection. It is difficult to correlate the size of the cyst to the degree of venous compression, but the location of the cyst shows to be an important contributor to venous compression. As shown in Table 1, the cyst was immediately posterior to the common femoral vein only in those patients with lower limb edema.

The follow-up of all cases was uneventful, and no recurrence appeared at 2 months post-surgery, some of these patients have significantly longer follow-up of 2 years, 4 years, 6 years and 10 years, respectively.

## Discussion

In all 7 cases of symptomatic synovial cyst in our retrospective study, surgical excision showed a favorable short-term or long-term prognosis. Symptoms, which were inguinal masses with or without edema in our study, were completely relieved within one week post-surgery, and the recurrence rate was zero.

Lower limb edema caused by synovial cyst of the hip joint is a rare occurrence. To date, there are less than 40 reported cases in the literature [2]. Melamed et al. reported a case in which the patient’s external iliac vein was compressed by an iliopsoas bursal extension of the arthritic hip joint [3]. Colasanti et al. reported a new case and summarized a total of 27 cases [4]. In their summary, the external iliac and femoral veins were most frequently involved, and 55% of the patients had a hip disorder, of which osteoarthritis and rheumatoid arthritis were the most common. They also proposed that those cases of “idiopathic” synovial cysts of the hip joint were due to subtle congenital and developmental defects that disrupted the dynamics of synovial fluid.

In addition to edema, synovial cyst of the hip joint can cause other problems including peripheral nerve compression, or even urinary symptoms [5, 6]. Nerve compression most commonly involves the femoral nerve. Lavyne et al. first reported a case of femoral neuropathy caused by an iliopsoas bursal cyst [7]. In cases with nerve compression, the cyst was often located anterior to the hip joint, and the symptoms mimicked radicular pain resulting from an L2–4 lumbar disorder. The sciatic nerve is the second most commonly compressed nerve; the first case was reported in 1991 by Juglard et al. [8], and symptoms were similar to sciatica. The obturator nerve and lateral cutaneous femoral nerve are much less frequently involved with few case reports [9–12].

In some cases, the symptomatic synovial cysts merely manifested as an inguinal mass, or pain and swelling in the inguinal area, as in cases 5 and 6 in our study, where the cysts grew superficially.

Although synovial cyst of the hip joint is a clinical rarity, advances in imaging technology make diagnosis more effective and less invasive. Ultrasound is a useful tool in evaluating the degree of venous stenosis and ruling out deep vein thrombosis as a common cause of lower limb edema. On ultrasound, a synovial cyst should appear as a hypoechoic lesion near the hip joint. Radiography can help to recognize hip disorders like osteoarthritis and rheumatoid arthritis. Duplex ultrasound can exclude femoral aneurysm as a possible reason for venous compression. Contrast-enhanced CT can show characteristic rim enhancement of the cyst. MRI, a superior imaging technique for its high resolution of soft tissue, can demonstrate the exact location and extent of the cystic lesion.

Multiple treatment options are available for synovial cyst of the hip joint, depending on the size of the cyst, the severity of symptoms, the primary disease or the comorbidities, and the severity of the local compression. Asymptomatic cysts should be under regular observation, and treatment for the primary disease is the priority. Studies show that if the primary disease is rheumatoid arthritis, treatment for RA reduces the size of the cyst [13, 14]. For symptomatic synovial cysts, radical treatments including surgical excision, which lowers recurrence rate [15], or needle aspiration are recommended. Because a synovial cyst is simply an enlarged iliopsoas bursa, puncture of the cyst can result in a fast refill, but no large-volume data have been collected to guide clinical management. Furthermore, it has been reported that injection of steroids or sclerosing agents can be effective [10, 16, 17].

## Conclusion

Our retrospective study shows that for symptomatic synovial cyst of the hip joint, surgical excision could successfully resolve the symptoms and no recurrence appeared at 2 months post-surgery of all cases, some of the patients had significantly longer follow-up of several years. We found that, instead of the size of the cyst, the location of the cyst was an important contributor to venous compression. Until more evidence is collected to generate a comprehensive management plan, surgical excision remains a well-established and well-accepted treatment for synovial cysts originating from the hip joint. Due to the small sample size, our conclusions are preliminary, and more evidence are required to establish a guideline for the diagnosis and treatment for synovial cyst of the hip joint.
